# Use of ICDAS-II, Fluorescence-Based Methods, and Radiography in Detection and Treatment Decision of Occlusal Caries Lesions: An In Vitro Study

**DOI:** 10.1155/2012/371595

**Published:** 2012-08-29

**Authors:** Anahita Jablonski-Momeni, Jasmin Stucke, Torben Steinberg, Monika Heinzel-Gutenbrunner

**Affiliations:** Department of Paediatric and Community Dentistry, Dental School, Philipps University of Marburg, Georg-Voigt Street 3, 35033 Marburg, Germany

## Abstract

*Aim*. To use visual inspection (ICDAS-II), laser fluorescence (LF), fluorescence based camera (FC) and radiographic examination (BW) for detection of caries and for treatment decision. *Methods*. The occlusal sites of 84 extracted permanent teeth were examined using all methods and treatment decisions (preventive or operative care) were recorded based on each method independently. For validation of the findings, fissures were opened with rotating instruments and clinical depth was determined as gold standard. Correlations (*r*_*s*_), sensitivity, specificity and AUC were calculated. McNemar test was used to show whether different methods led to significant changes in treatment decisions. *Results*. Highest correlation was found between ICDAS-II and FC (*r*_*s*_ 0.84), ICDAS-II and gold standard (0.82) and FC and gold standard (0.81). ICDAS-II provided the highest performance (AUC 1.0), followed by FC (0.95) and LF (0.88). The greatest difference was found for treatment planning of dentine lesions, where the use of FC (cut-offs according to the literature) had the greatest agreement between operative treatment and dentine lesions, followed by use of ICDAS-II. *Conclusion*. ICDAS-II may have high potential for detection and treatment planning, and other devices, especially the fluorescence camera, can add substantial information to the visual examination, enabling examiners plan treatment more accurately.

## 1. Introduction

Apart from thorough detection and diagnosis of dental caries, compiling a treatment plan is another important task of a dentist. The initial assessment of hard tooth tissue is normally visual and, depending on indication and availability, X-rays are used for further detection and treatment planning.

The visual classification system International Caries Detection and Assessment System (ICDAS-II) was developed to provide clinicians, epidemiologists, and researchers with an evidence-based method for standardized data collection in different settings and better comparison between studies [1]. Reproducibility and accuracy of ICDAS-II have already shown to be promising for occlusal caries detection [2]. ICDAS criteria have the potential to aid treatment planning [3]. Depending on the visual assessment, activity of a lesion, and patient's risk status, the preferred care options might be tilted towards preventive or operative treatment.

Apart from purely visual and visual-tactile caries diagnosis, there are several other methods for the detection of dental caries on occlusal surfaces. This includes radiography, laser or light fluorescence-based methods, and electrical impedance measurements. It is well known that fluorescence-based methods make use of the phenomenon that carious lesions fluoresce more strongly than sound tissues when excited by light at specific wavelengths. The devices DIAGNOdent and DIAGNOdent pen (KaVo, Biberach, Germany) function on the same principle: they emit red light at 655 nm that causes fluorescence of bacterial metabolites in infected dentine [6]. The fluorescence emitted from the tooth is measured and translated into a numerical scale from 0 to 99. The VistaProof fluorescence camera and the recently devised VistaCam iX (both: Dürr Dental, Bietigheim-Bissingen, Germany) have LEDs that emit high-energy blue-violet light at 405 nm onto the tooth surface. This wavelength stimulates porphyrins produced by caries-related bacteria to emit red light, containing less energy. Sound enamel, in contrast, sends out green light. This fluorescence is recorded by the camera, transferred to a computer, and processed with special software (DBSWIN, Dürr). The result is a digital image that shows lesions in different colors with respective numerical values between 0 and 4, predicting the extent and depth of caries [5, 7–9].

Radiographic examination is quite commonly used in caries detection and generally it is possible to detect approximal lesions earlier than with visual diagnosis alone [10]. However, the validity of detecting enamel caries is low for the occlusal surfaces [11]. 

To date, only a few studies exist which look at the impact of using visual inspection, radiographic examination, or laser fluorescence devices in treatment decision [12, 13]. No study to date has looked at the impact of the use of the new VistaCam iX fluorescence device in treatment decisions. Thus the aim of this study was to evaluate the performance of visual ICDAS-II, laser fluorescence, fluorescence-based camera, and radiographic (BW) examinations for occlusal caries detection and their ability to make treatment decisions when used as a single method or as a combination of two methods.

## 2. Material and Methods

### 2.1. Sample Selection and Visual Examination

Eighty-four permanent posterior teeth without occlusal restorations were available for the study. The teeth were collected in a dental practice and informed consent was obtained for the use of teeth for scientific purposes. The teeth were stored in a thymol-water solution immediately after extraction. Within 24 h, they were cleaned thoroughly and then stored in water. The occlusal surfaces were photographed digitally (Leica Zoomsystem Z6 APO/QWin Standard V 3.4.0 software, Leica Microsystems, Wetzlar, Germany). One site within the pit and fissure system of each tooth was marked on black and white images of the tooth surface for ease of relocation.

All sites were visually examined by two investigators (doctoral student calibrated by an experienced investigator) using the International Caries Detection and Assessment System (ICDAS-II) [14] and a consensus score for each site was achieved. The chosen sites were recorded as:

 0 = sound (*n* = 13);
 1 = first visible sign of noncavitated lesion seen only when the tooth is dried;
 2 = visible noncavitated lesion seen when wet and dry;
 3 = microcavitation in enamel;
 4 = noncavitated lesion extending into dentine seen as an undermining shadow;
 5 = small cavitated lesion with visible dentine: less than 50% of surface;
 6 = large cavitated lesions with visible dentine in more than 50% of the surface.

### 2.2. Examination of Occlusal Surface with the DIAGNOdent Laser Fluorescence Device (LF) and the VistaCam iX Fluorescence-Based Camera (FC)

Prior to the evaluations, the examiners were trained in using the devices according to the manufacturer's recommendations. The DIAGNOdent laser fluorescence device (LF) was first calibrated using a ceramic standard as suggested by the manufacturer. Prior to the laser fluorescence readings, the device was zeroed using an obviously sound enamel spot on the tooth (zero value). This overcomes inherent differences in tooth color. Using tip A, the laser fluorescence device was moved along the surface of the investigation site and the peak value was recorded (possible reading values 0–99). Using the VistaCam iX fluorescence-based camera (FC), images of all occlusal tooth surfaces were taken.For each tooth, the distance spacer was used in order to achieve optimum results with the caries filter. The optical head of the camera was placed on the occlusal surface of the tooth and the control ring was pressed in order to freeze the image taken by the camera. The saved images were analyzed by the camera software (DBSWIN, Dürr Dental) with possible reading values from 0–4. Detailed information on the scales of both LF and FC is given in Tables 1 and 4.

For both fluorescence methods, the sites were assessed independently from each other. Intraexaminer reproducibility for both devices was assessed by repeating the measurements on 1/3 of the investigation sites (*n* = 28) within 1 day.

### 2.3. Examination of Bitewing Radiographs (BW)

Digital BW radiographs were taken of all teeth using the Gendex dental X-ray machine (Soredex, Helsinki, Finland) at 65 kV, 6.5 mA, and exposure time of 0.12 seconds. All radiographs were processed by the Digora Optime image scanner (Soredex, Helsinki, Finland). The teeth were placed in rows (3 teeth in each row) in suitable molds (A-R X-ray model, frasaco GmbH, Tettnang, Germany).

The digital bitewings were then viewed on an 18 in. TFT (thin-film transistor) color monitor (FlexScan L 768, EIZO, Avnet Technology Solutions GmbH, Nettetal, Germany) by the investigators and a consensus score for each site was achieved. The sites were recorded using the following scores:

 0 = no radiolucency,
 1 = radiolucency in the outer half of enamel,
 2 = radiolucency in the inner half of enamel, up to the enamel-dentine junction,
 3 = radiolucency in the outer half of dentine,
 4 = radiolucency in the inner half of dentine.

### 2.4. Determination of Treatment Decision

For all sites a consensus treatment decision was made by the examiners using the following scores: 0 = no treatment/use of fluorides, 1 = sealant application, 2 = round bur and sealant application, and 3 = restoration (e.g., composite, amalgam, and glass ionomer). For further analysis the treatment decisions were collapsed into preventive care (pc: score 0-1) and operative care (oc: score 2-3). 

### 2.5. Determination of Lesions' Depth (Gold Standard)

To determine the depth of the lesions, all tooth surfaces were opened with rotating instruments. The end point of the excavation was reached when there was no longer any sign of caries [15]. In the process, the lesions' depths were divided up into the following categories: sound tooth surface (score 0), caries in the enamel (clinical score 1), caries down to the enamel-dentine junction (clinical score 2), and caries in the first third of dentine (clinical score 3), caries down to the second third of dentine or near pulp, or pulp already effected (clinical score 4).

All the sites were investigated by two examiners and a consensus clinical score was derived for each investigation site.

### 2.6. Data Management and Statistical Evaluation

All findings were recorded on data collection forms and later transferred to an Excel table. The statistical analysis was performed using MedCalc, Version 11.3.4.0. Intraexaminer reproducibility was calculated using the intraclass correlation coefficient (ICC). The relationship between all the systems was assessed using the Spearman rank correlation.

The consensus clinical scores were used to calculate sensitivity and specificity at the D_1_ and D_3_ diagnostic threshold. At the D_1_ diagnostic threshold all clinical scores 1–4 were classed as caries, for the D_3_ diagnostic threshold clinical scores 3 and 4 were classed as caries only. Using these sensitivity and specificity values ROC (receiver operating characteristic) analyses were carried out for each method. Sensitivity and specificity values for the LF measurements were obtained at the cut-off values according to the literature [4]. For the FC, sensitivity and specificity values were used firstly at the cut-off values according to the manufacturer and secondly at the cut-off values which were determined according to the literature [5]. The performance of each method (AUC) was interpreted by using the following classification: 0.50–0.60 fail, 0.60–0.70 poor, 0.70–0.80 fair, 0.80–0.90 good, and 0.90–1.0 excellent [16, 17]. 

A nonparametric test (the McNemar test) was used to show whether different methods or combinations of methods lead to significant changes in treatment decisions (*α* = 0.05).

## 3. Results

A total of 84 occlusal sites on posterior teeth were examined (52 molar and 32 premolar teeth). The distribution of the sites according the ICDAS-II criteria was code 0 = 13, code 1 = 15, code 2 = 26, code 3 = 12, code 4 = 2, code 5 = 12, and code 6 = 4. 

While using the FC device, 4 investigation sites could not be assessed due to technical problems. Thus, further statistical calculation for the FC was performed using 80 sites. Intraclass correlation coefficients (ICC) for intraexaminer reproducibility were 0.98 for the LF device and 0.97 for the FC.

The distribution of the sites according to the different methods cross-tabulated with the gold standard scores is presented in Table 1. All methods assessed nearly all sound tooth surfaces correctly, except for FC when the cut-off values according to the manufacturer were used.

Spearman correlation coefficients between all the methods are presented in Table 2. All correlations were significant at the 0.01 level or at the 0.05 level, respectively (two tailed). The highest correlation was found between ICDAS-II and FC measurements followed by the correlation between ICDAS-II and the gold standard. The lowest correlation was found between the findings of bitewing radiography and the gold standard.

Sensitivity, specificity, and the areas under the ROC curves (AUC) at D1 and D3 diagnostic thresholds are presented in Table 3. The use of ICDAS-II, LF, and FC demonstrated good to excellent diagnostic performances (AUC between 0.88 and 1.0), while the performance of bitewing radiography for detection of occlusal lesions was shown to be weak (AUC 0.56 and 0.59, resp.). At the D1 diagnostic level specificity for the FC was low using the cut-off suggested by the manufacturer. When other cutoffs [5] were used, specificity increased to 100%. The same findings were observed for the sensitivity of the FC at D3 diagnostic level.

Table 4 presents the distribution of the sites according to the different methods cross-tabulated with the treatment decision.  Table 5 shows whether the treatment decisions changed significantly from preventive care to operative care when different methods were used for treatment planning. For example, when treatment decisions with ICDAS-II were cross-tabulated with treatment decisions after bitewing assessment, operative rather than preventive treatment was chosen for significantly more sites (*n* = 19 and *P* = 0.003). Similar findings were observed for treatment decisions involving ICDAS-II versus FC with regular cutoffs (*P* < 0.001), LF versus FC (*P* < 0.001), or LF versus BW (*P* = 0.019). When the FC findings were classified according to the cutoffs in the literature [5], the treatment decisions shifted significantly toward the operative approach (*P* < 0.001).

In Table 6 the treatment decision after combining each method with the visual system ICDAS-II is cross-tabulated with the treatment decision of the method alone. It can be seen that the best agreement was found when the treatment decision was based on a combination of ICDAS-II and FC (cutoffs set by the manufacturer) compared to ICDAS-II-based decisions alone, followed by a combination of ICDAS-II and bitewing radiography.

In Table 7 the results of the treatment decision according to different methods and the gold standard are cross-tabulated. It can be seen that non-operative care for sound surfaces was suggested with each method, indicating high specificity of the treatment decision. With the FC the decision for operative treatment was made for 10 surfaces with caries only in the enamel, when cutoffs according to the literature were used. The corresponding numbers for the other methods were much lower (between 0 to 4 surfaces with enamel lesion). On the other hand, almost all of the clinically dentine lesions were planned to be treated operatively (34/36).

## 4. Discussion

Ideal management of a caries lesion includes not only the use of criteria for extent of lesion but also treatment planning which should express the results of lesion assessment in terms of background lever care, preventive treatment options, and operative treatment options [18]. The specific treatment options recommended for specific lesions and patients will depend upon a variety of factors, such as lesion activity and monitoring lesion behavior over time [18]. Of course, these factors cannot be considered in an in vitro study such as this, but only factors such as lesion depth and extent, color, and the tooth surface where the lesion is assessed. 

In the present study, occlusal lesions were determined using various currently common methods of caries detection and treatment options recommended on the basis of these examinations. In the process, methods of fluorescence and laser fluorescence were given particular attention. The results show that the visual method and the fluorescence camera exhibited the highest correlation to the gold standard (Table 2). By contrast, caries detection using bitewing radiographs showed the lowest correlation, as well as the lowest diagnostic quality with regard to the detection of occlusal lesions (Table 3). 

Modern dentistry now offers many options for preventive treatment (e.g., the use of fluorides or other remineralizing agents, fissure sealing, caries infiltration, etc.). In order to make such decisions, however, we need to carefully detect healthy hard tooth tissue as well as initial lesions so that they too can be given preventive therapy. The visual ICDAS-II and apparatus-based (LF and FC) methods used here have shown their potential for discovering and differentiating a range of carious lesions in a variety of studies [2, 5, 19].

The present study investigated whether each of these means of detection alone would be capable of indicating an adequate treatment recommendation with regard to a preventive or operative treatment option when the recommended or already published cutoffs were used to differentiate healthy teeth, enamel lesions, and dentine caries. Since the reproducibility of the fluorescence systems in particular is very high, it should be assumed that the detection of the lesions, and thus the therapy decision would be possible after using the method several times (monitoring) as well as if the attending dentist should change. In this study, the ICC values for the LF and FC were high for intraexaminer reproducibility and the figures were comparable to studies already published [5, 20]. The reproducibility of ICDAS-II of one of the reference examiners in this study was the subject of other studies, where intraexaminer kappa values were in a range of 0.82–0.93 [2, 21, 22]. In the present study, a consensus decision was made for the ICDAS-II, and hence no reproducibility values were calculated.

The analysis of AUC, sensitivities, and specificities at different diagnostic thresholds (Table 3) showed that ICDAS-II had excellent AUC as well as high sensitivity and specificity. Initial in-vitro studies using ICDAS-II for detection of occlusal lesions [2, 7] showed lower overall performance of ICDAS-II (AUC between 0.73–0.88). Later studies by some authors of the present study clearly showed an increase of ICDAS-II performance, sensitivity, and specificity values [15, 22] which might be explained by the enhanced training in this system. Comparing the performance of the fluorescence systems with other studies, it can be shown that the reported figures stay constant. For example, the performance of laser fluorescence measurements was reported to be between 0.66–0.86 in different studies and under different conditions [7, 20, 23]. The performance of the newly introduced VistaCam iX fluorescence camera has not yet been the subject of many studies. Jablonski-Momeni et al. [5] calculated AUC between 0.87 and 0.92 for 2 examiners at the D1 and D3 diagnostic threshold. The authors showed that there was no significant difference to the performance of the already well-known VistaProof. The performance of this device ranged between 0.75 and 0.96 in different studies [5, 7, 24] and would thus seem to be a useful tool for the detection of occlusal lesions. However, the good performance of the apparatus-based procedure must be seen in a somewhat differentiated way: different cut-off points were predefined by the manufacturer at the launch of the VistaCam iX camera. These cut-off points were intended to facilitate differentiation of the caries stages from sound to dentine caries with the help of the numerical scale. According to this, values ≥1.0 should be considered proof of the beginning of enamel caries, while dentine caries supposedly produced values ≥2.0. Previous studies [5] of the optimum sensitivity and specificity have shown that this cut-off point should be set at 1.4/1.5 to detect dentine lesions appropriately, which means that lesions >1.5 can be expected to indicate dentine caries. A comparable cut-off point (>1.4) for the determination of dentine caries was found in other studies using the VistaProof [9, 24, 25]. In the present study, treatment decisions were determined using the cut-off points according to the manufacturer and according to the literature [5], respectively. It could be seen that sensitivity values at the D1 and D3-level were higher when cutoffs according to the literature [5] were used (Table 3). When these cutoffs were used for treatment planning, almost all of the clinically dentine lesions were planned to be treated operatively (34/36, Table 7). However, it should be taken into account that 4 investigation sites could not be assessed due to technical problems while using the FC for caries detection.

The use of BW for occlusal caries detection showed low sensitivity (Table 3). Other authors had reported higher values of sensitivity but similar specificity values when BW radiographs were evaluated for occlusal caries detection [26, 27]. Looking at the correlation coefficients found in our study (Table 2) it can be observed that BW had the weakest correlation compared to all other methods and to the gold standard, respectively. When BW was used for treatment decisions, more than 70% (29/40, Table 7) of clinically dentine lesions were planned to be treated in a preventive manner. Usually studies on using radiography and treatment decisions tend to use BW for approximal caries detection rather than for occlusal lesions [28]. Diniz et al. [13] suggested in a recently published study that the ICDAS examination shows better performance than radiographic examination for occlusal caries detection. These findings were confirmed by the present study.

In vitro studies usually establish the validity of a detection system by using a gold standard against which the sensitivity and specificity of the diagnostic method can be calculated. A common gold standard used for occlusal caries lesions is the histological evaluation of hard tissue sections, with large variations in the methodology. Teeth are hemisected at the place to be examined, or hard tissue sections are prepared whose thickness can vary considerably [29]. In order to be close to clinical conditions we determined the depth of the lesions by opening the tooth surfaces with rotating instruments. This method might show some shortcomings, and in order to validate whether the caries was removed in total sectioning of the excavated teeth could probably have been an additional option. 

The results of our study show that preventive care for sound surfaces was suggested with each detection method, indicating high specificity of the treatment decision (Table 7). Almost all enamel lesions which were found clinically were planned to be treated using preventive treatment options. The greatest difference was found for treatment planning of dentine lesions, where the use of FC (with cut-offs according to the literature) had the greatest agreement between operative treatment and dentine lesions, followed by use of ICDAS-II. This information may suggest that the use of FC is the primary criterion for evaluating the condition of the dental surface before the establishment of a treatment plan, followed by ICDAS-II. But it should be taken into account that use of detection tools should always be seen as an adjunct to a primary visual detection of dental caries and thus it should rather be the other way round: that a tooth surface is first observed by a visual detection method and then as a second step, another device can be used for further detection. Treatment options can be suggested as a consequence of the examinations. 

Ie and Verdonschot [30] observed that although carious lesions can be observed by visual examination, caries preventive strategies will not be initiated until very late. A solution to this problem would be to detect caries lesions at an early stage of development, for example, by using systems like ICDAS-II. In the study by Diniz et al. [13] ICDAS has proved to be reliable for early caries detection, but there was no strong correlation with histology. In the present study the findings (correlation to gold standard, performance, sensitivity, and specificity values) indicate that ICDAS-II may have a high potential for detection and treatment planning, and that other devices, especially the fluorescence camera, could add substantial information to the visual examination, enabling examiners to plan treatment more accurately.

Of course, issues like caries activity and patient-based information cannot be taken into account for treatment decisions within the limitations of an in vitro study.

## Figures and Tables

**Table 1:** Cross-tables showing the relationship between the different methods and the gold standard.

	Gold standard (clinical lesion depth)					
	0	1	2	3	4	Total
ICDAS-II scores						
0 (sound)	13	0	0	0	0	13
1-2 (enamel lesion)	0	13	16	8	4	41
3–6 (dentine lesion)	0	0	2	9	19	30

Total	13	13	18	17	23	84

LF scores*						
0–7 (sound)	12	9	10	2	1	34
8–24 (enamel lesion)	1	2	6	9	6	24
25–99 (dentine lesion)	0	2	2	6	16	26

Total	13	13	18	17	23	84

FC scores**						
0–0.9 (sound)	1	0	0	0	0	1
>0.9–2 (enamel lesion)	12	13	18	16	13	72
>2 (dentine lesion)	0	0	0	1	6	7

Total	13	13	18	17	19	80

FC scores***						
0–1.2 (sound)	13	8	4	0	0	25
1.3–1.4 (enamel lesion)	0	4	5	2	0	11
>1.4 (dentine lesion)	0	1	9	15	19	44

Total	13	13	18	17	19	80

Bitewing scores						
0 (sound)	12	12	16	16	13	69
1-2 (enamel lesion)	0	0	0	0	0	0
3-4 (dentine lesion)	1	1	2	1	10	15

Total	13	13	18	17	23	84

*Cutoffs according to the literature [4].

**Cutoffs according to the manufacturer's recommendation.

***Cutoffs according to the literature [5].

**Table 2:** Spearman's correlation coefficients between different methods.

	Spearman's correlation coefficient (*r*_*s*_)			
	LF	FC	BW	Gold standard
ICDAS-II	0.66**	0.84**	0.36**	0.82**
LF	—	0.72**	0.27*	0.69**
FC	—	—	0.26*	0.81**
BW	—	—	—	0.22*

*Correlation significant at the 0.05 level (two tailed).

**Correlation significant at the 0.01 level (two tailed).

**Table 3:** The area under the ROC curve (AUC), sensitivity (SE), and specificity (SP) for each method at the D_1_ and D_3_ diagnostic thresholds.

D_1_ diagnostic threshold	ICDAS-II	LF	FC		BW
AUC (95% CI)	1.0 (0.96–1.0)	0.88 (0.79–0.94)	0.95 (0.88–0.99)		0.56 (0.44–0.67)
SE (%)	100	69.0*	100**	82.1***	19.7
SP (%)	100	92.3*	7.7**	100***	92.3

D_3_ diagnostic threshold	ICDAS-II	LF	FC		BW

AUC (95% CI)	0.92 (0.84–0.97)	0.88 (0.79–0.94)	0.93 (0.85–0.98)		0.59 (0.48–0.67)
SE (%)	70.0	52.5*	19.44**	94.4***	27.5
SP (%)	95.5	90.9*	100**	77.3***	90.9

CI: confidence interval.

*Cutoffs according to the literature [4].

**Cutoffs according to the manufacturer's recommendation.

***Cutoffs according to the literature [5].

**Table 4:** Cross tables showing the relationship between the different methods and the treatment decisions.

	Preventive care	Operative care	Total
ICDAS-II scores			
0 (sound)	13	0	13
1-2 (enamel lesion)	41	0	41
3–6 (dentine lesion)	0	30	30

Total	54	30	84

LF scores*			
0–7 (sound)	34	0	34
8–24 (enamel lesion)	24	0	24
25–99 (dentine lesion)	0	26	26

Total	58	26	84

FC scores**			
0–0.9 (sound)	1	0	1
>0.9–2 (enamel lesion)	72	0	72
>2 (dentine lesion)	0	7	7

Total	73	7	80

FC scores***			
0–1.2 (sound)	25	0	25
1.3-1.4 (enamel lesion)	11	0	11
>1.4 (dentine lesion)	0	44	44

Total	36	44	80

Bitewing scores			
0 (sound)	69	0	69
1-2 (enamel lesion)	0	0	0
3-4 (dentine lesion)	0	15	15

Total	69	15	84

*Cutoffs according to the literature [4].

**Cutoffs according to the manufacturer's recommendation.

***Cutoffs according to the literature [5].

**Table 5:** Cross tables showing the relationship between the treatment decisions when different methods were used.

Treatment decision after using	Number of teeth related to the treatment decision							
	LF*		FC**		FC***		BW	
ICDAS-II	pc	oc	pc	oc	pc	oc	pc	oc

pc	45	9	54	0	34	20^a^	50	4
oc	13	17	19^a^	7	2	24	19^b^	11

LF*			pc	oc	pc	oc	pc	oc

pc			57	1	34	24^a^	54	3
oc			16^a^	6	2	20	15^c^	11

FC**					pc	oc	pc	oc

pc					36	37^a^	65	8
oc					0	7	3	4

FC***							pc	oc

pc							33	3
oc							35^a^	9

pc: preventive care, oc: operative care.

*Cutoffs according to the literature [4].

**Cutoffs according to the manufacturer's recommendation.

***Cutoffs according to the literature [5].

Within columns, significant differences are represented by different superscript letters (the McNemar test: a: *P* < 0.001/b: *P* = 0.003/c: *P* = 0.019).

**Table 6:** Cross tables showing the relationship between the treatment decisions when each method was combined with the visual inspection.

Treatment decision after using	Number of teeth related to the treatment decision							
	ICDAS-II + LF*		ICDAS-II + FC**		ICDAS-II + FC***		ICDAS-II + BW	
ICDAS-II	pc	oc	pc	oc	pc	oc	pc	oc

pc	45	9^a^	54	0	34	20^b^	50	4
oc	0	30	0	30	0	30	0	30

LF*	pc	oc						

pc	45	13^b^						
oc	0	26						

FC**			pc	oc				

pc			54	19^b^				
oc			0	7				

FC***					pc	oc		

pc					34	2		
oc					0	44		

BW							pc	oc

pc							50	19^b^
oc							0	15

pc: preventive care, oc: operative care.

*Cutoffs according to the literature [4].

**Cutoffs according to the manufacturer's recommendation.

***Cutoffs according to the literature [5].

Within columns, significant differences are represented by different superscript letters (the McNemar test: a: *P* = 0.004/b: *P* < 0.001).

**Table 7:** Cross tables showing the relationship between the treatment decision according to different methods and the gold standard.

Treatment decision after	Gold standard (clinical lesion depth)			
	0 (sound)	1-2 (enamel lesion)	3-4 (dentine lesion)	Total
ICDAS-II				
pc	13	29	12	54
oc	0	2	28	30

Total	13	31	40	84

LF*				
pc	13	27	18	58
oc	0	4	22	26

Total	13	31	40	84

FC**				
pc	13	31	29	73
oc	0	0	7	7

Total	13	31	36	80

FC***				
pc	13	21	2	36
oc	0	10	34	44

Total	13	31	36	80

Bitewing				
pc	12	28	29	69
oc	1	3	11	15

Total	13	31	40	84

pc: preventive care, oc: operative care.

*Cutoffs according to the literature [4].

**Cutoffs according to the manufacturer's recommendation.

***Cutoffs according to the literature [5].
